# Mechanism of Strength Formation of Unfired Bricks Composed of Aeolian Sand–Loess Composite

**DOI:** 10.3390/ma17051184

**Published:** 2024-03-04

**Authors:** Deren Liu, Yafang Guo, Yanjie Zhang, Zhechao Zhu, Pengju Xu, Shize Zhang, Yugang Ren

**Affiliations:** 1 School of Civil Engineering, Lanzhou Jiaotong University, Lanzhou 730070, China; liuderen@mail.lzjtu.cn (D.L.); shuizhongdelantian@163.com (Y.Z.); 13511359421@163.com (Z.Z.); 15117132748@163.com (P.X.); zsz2322883839@163.com (S.Z.); 18009381464@163.com (Y.R.); 2 National and Provincial Joint Engineering Laboratory of Road & Bridge Disaster Prevention and Control, Lanzhou 730070, China

**Keywords:** aeolian sand, loess, unfired brick, physical properties, microstructure, durability

## Abstract

Aeolian sand and loess are both natural materials with poor engineering-related properties, and no research has been devoted to exploring aeolian sand–loess composite materials. In this study, we used aeolian sand and loess as the main raw materials to prepare unfired bricks by using the pressing method, along with cement, fly ash, and polypropylene fiber. The effects of different preparation conditions on the physical properties of the unfired bricks were investigated based on compressive strength, water absorption, and softening tests and a freeze–thaw cycle test combined with X-ray diffraction and scanning electron microscope analysis to determine the optimal mixing ratio for unfired bricks, and finally, the effects of fibers on the durability of the unfired bricks were investigated. The results reveal that the optimal mixing ratio of the masses of aeolian sand–loess –cement –fly ash–polypropylene fiber–alkali activator–water was 56.10:28.05:9.17:2.40:0.4:0.003:4.24 under a forming pressure of 20 MPa. The composite unfired bricks prepared had a compressive strength of 14.5 MPa at 14 d, with a rate of water absorption of 8.8%, coefficient of softening of 0.92, and rates of the losses of frozen strength and mass of 15.93% and 1.06%, respectively, where these satisfied the requirements of environmentally protective bricks with strength grades of MU10–MU15. During the curing process, silicate and sodium silicate gels tightly connected the particles of aeolian sand and the loess skeleton, and the spatial network formed by the addition of the fibers inhibited the deformation of soil and improved the strength of the unfired bricks.

## 1. Introduction

By 2023, the expanse of desert regions in China reached an area of 3.309 million square kilometers, constituting 34.56% of the nation’s terrestrial territory, predominantly situated in the northwestern and northern provinces [1]. The sandy and windy environmental conditions prevalent in these locales exert a profound impact on diverse sectors including agriculture, livestock rearing, ecological preservation, and the integrity of infrastructural constructs [2,3,4], such as, notably, the Tengger and Mao Wusu Deserts, located in the northwestern part of China, as well as the Loess Plateau, an area renowned for its unique blend of aeolian sand and loess deposits [5]. The granular makeup of aeolian sands is characterized by their homogeneity in particle size, heightened porosity, and notably deficient cohesion and shear resistance [6,7]. The inherent macrostructure of the loess is such that it is prone to dramatic reductions in mechanical strength and concurrent escalations in deformation when subjected to aqueous conditions or high humidity, leading to a propensity for collapses [8,9,10]. Due to the special nature of aeolian sand and loess, their properties need to be improved to meet the requirements of engineering construction.

Previous studies have shown that the addition of cement, lime, and other cementing materials to aeolian sand can improve the compactness of aeolian sand and reduce its porosity, thus improving its compressive, shear, and tensile strength [6,11,12,13,14,15,16,17]. At present, aeolian sand has been widely used in the preparation of mortar, concrete, and roadbed filler [18]. In addition, many scholars have found that the sand can be improved by adding solid waste, fibers, etc. Arias-Trujillo et al. [19] used ceramic brick waste aggregate and cement to improve the mechanical properties of sand, including compressive strength, California bearing ratio, etc. Yang et al. [20] used polypropylene fibers, silt, and cement to improve the mechanical properties of aeolian sand, combined with compressive strength tests and microscopic means, and analyzed the curing mechanism of aeolian sand. In addition, there are some studies related to loess improvement [21,22,23]. Xue et al. [21] developed a cementing material by using industrial waste, used the material to cure loess, and found that the unconfined compressive strength and durability of cured loess were significantly improved, and the strength was mainly derived from the hydration products of the cementing material. Yang et al. [23] conducted a study on silica micro powder and cement-curing loess through indoor tests, and the results showed that silica micro powder mixed with cement could effectively improve the mechanical properties of natural loess through hydration and durability.

With the implementation of China’s western development, more and more infrastructures are being built in China’s desert areas as well as the Loess Plateau, and at the same time, there is a growing demand for construction materials, which also includes brick materials [24]. Traditional sintered bricks have the disadvantages of high energy consumption and substandard carbon emissions. To transform sintered bricks for energy saving and consumption reduction, some scholars have conducted relevant research on the green transformation of traditional sintered wall materials, focusing on the development of clay-free, energy-saving, carbon-reducing, eco-friendly, sinter-free new wall materials [25,26,27,28,29]. Several studies have demonstrated that industrial by-products can be used to produce unfired bricks, which have remarkable strength and durability due to the formation of cementing compounds such as hydrated calcium silicate (C-S-H) and hydrated calcium silicate aluminate (C-S-A-H) gels [18,30]. Dai et al. [30] investigated the pressing process of unfired bricks consisting of molybdenum tailings and cement, where the mass fraction of the cement was not less than 20%. This effect increased the density and strength of the unfired bricks. Some have prepared unfired bricks by replacing part of the clay with waste concrete and investigated the effects of different binders including cement and blast furnace slag on the non-shrinkage compressive strength and frost resistance of the unfired bricks [11,31].

Aeolian sand and loess are widely distributed [1,6,32]. If they are used as raw materials to produce unfired bricks, on one hand, this could indirectly suppress the process of desertification. On the other hand, this could maximize the effective utilization rate of aeolian sand and loess, alleviating the scarcity of resources. However, research on the preparation of composite unfired bricks from aeolian sand and loess is still relatively rare.

In this study, we investigated the feasibility of preparing unfired bricks using aeolian sand and loess based on the high-pressure pressing method. We used aeolian sand and loess as the main raw materials and supplemented them with cement, fly ash, and polypropylene fibers. We also systematically investigated the effects of different mixing ratios, forming pressures and curing ages on the compressive strength, water absorption, softening coefficient, and frost resistance of the unfired bricks. The results provide a reference for the use of aeolian sand and loess in construction.

## 2. Experimental Program

Before delving into the specifics of the experimental setup and methodology, it is imperative to delineate the context and the rationale behind the selection of materials and procedures employed in this study. The following segment of the paper meticulously outlines the materials sourced and the experimental framework designed to interpret the performance characteristics of unfired bricks formulated from aeolian sand–loess composite. This comprehensive approach ensures that the results garnered are robust and reproducible, contributing to the fundamental understanding and potential application of these materials in construction engineering.

### 2.1. Materials

**Figure 1:**
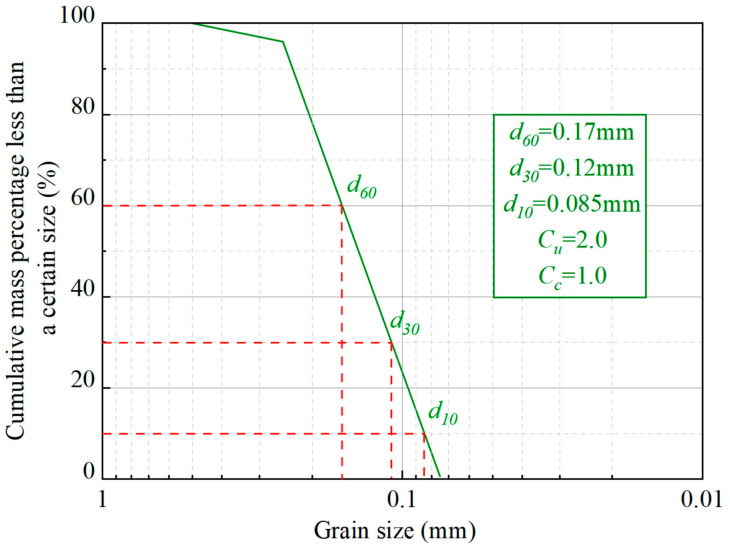
Sieving results of aeolian sand.

Aeolian sand was collected from the Tengger Desert in China, and was subjected to particle analysis, compaction tests, scanning electron microscopy (SEM), X-ray diffraction (XRD), and elemental composition analysis. The results are shown in Figure 1. The particle size of the sample ranged from 0.075 mm to 0.5 mm, which is similar to that in previous studies [33,34,35,36]. Its curves of gradation, d_10_ = 0.085, d_30_ = 0.12, and d_60_ = 0.17, as well as the coefficients of inhomogeneity Cu = 2.0 and curvature C_c_ = 1.0 showed that the particles of the sample were uniform, with poor gradation.

**Figure 2:**
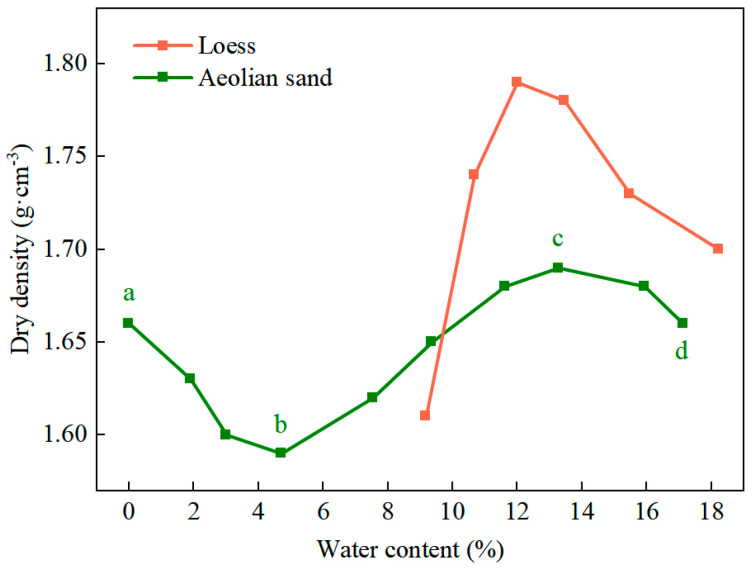
Curves of standard heavy compaction. a–d are characteristic points of the curve.

The curves of compaction of the aeolian sand and the loess obtained by using the standard heavy compaction test are shown in Figure 2. For the aeolian sand, the curve had a transverse “S” shape, and its dry density exhibited two peaks with a change in its water content: in the dry state with zero water content (point a) and in the saturated state with the optimal water content (point c). The dry density of the aeolian sand was 1.66 g/cm^3^ at point a, and a thin film of water was formed on the surface of the sand particles as the water content was increased from 0% to 4.8% (section ab). It had a high viscosity and cohesion, where this led to the difficulty of compaction between the particles of the sand such that its dry density decreased sharply and reached a minimum value of 1.59 g/cm^3^. As the water content was increased (section bc), the film of the water between the sand particles gradually thickened, their resistance to friction and cohesion decreased, the effect of lubrication increased, and the dry density increased continuously to reach a maximum value of 1.69 g/cm^3^ at the optimal water content. Once the water content had exceeded point c (section cd), the internal frictional resistance and cohesion of the sand particles continued to decrease. Furthermore, the incompressibility of water and its increasing volume caused a reduction in the dry density of the aeolian sand when subjected to identical compaction efforts.

**Figure 3:**
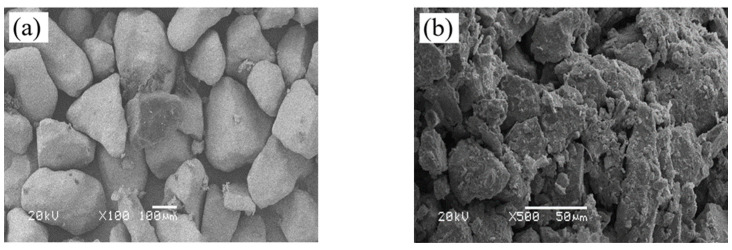
SEM photographs of (**a**) Aeolian sand and (**b**) loess.

Figure 3a shows SEM photographs of aeolian sand. The particles do not have prominent edges or smooth surfaces.

The loess was taken from the Lanzhou New Area, and Figure 2 shows that the optimal water content of the loess was 12% and its maximum dry density was 1.79 g/cm^3^. Figure 3b shows that most of the loess particles were granular and patchy, with a clear outline of the skeleton and prominent edges.

**Figure 4:**
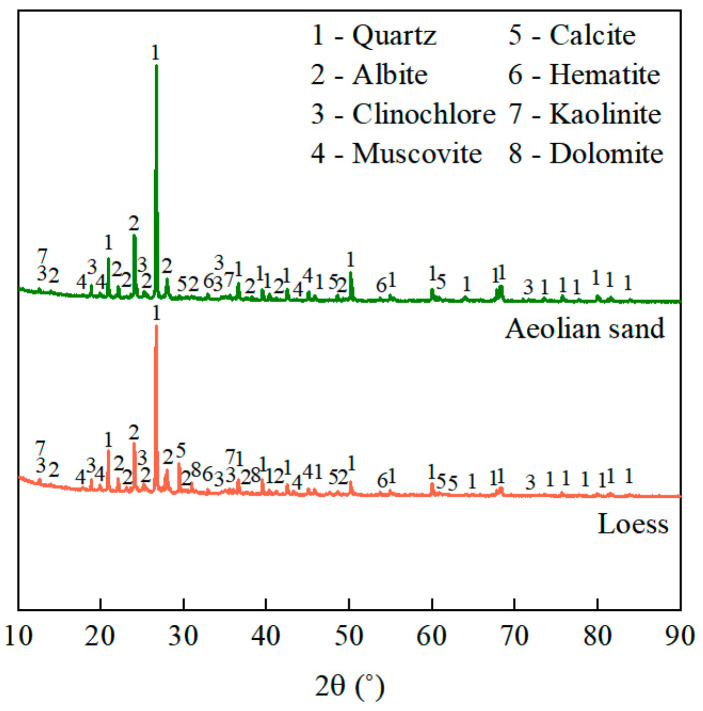
XRD patterns of aeolian sand and loess.

Data on the XRD spectra and chemical compositions of the aeolian sand and loess are listed in Figure 4 and Table 1, respectively. Their mineral and elemental compositions were approximately the same. However, as shown in Table 1, there are large differences in the chemical compositions of CaCO_3_ and CaMg(CO_3_)_2_ between aeolian sand and loess. The loess contains 10.7% of CaCO_3_ which, through hydration reactions, facilitates the formation of C-S-H, contributing to the enhanced strength of unfired bricks [37,38]. Additionally, the presence of CaMg(CO_3_)_2_, a hard chemical component found in loess, also contributes to the strength of unfired bricks [39,40]. Thus, considering only the chemical composition without taking into account factors such as grain size and structure, loess contributes more to the strength of unfired bricks than aeolian sand.

We used Ordinary Portland Cement with a strength grade of 42.5 (P.O 42.5 cement), manufactured by Conch Cement Co., Ltd. (Anhui, China), and commercially available primary fly ash with a density of 2.55 g/cm^3^. Their chemical compositions are shown in Table 2.

We also used bundled monofilament fibers composed of pure polypropylene, with diameters ranging from 18 to 48 μm and with a length of 6 mm.

Chemically pure sodium hydroxide was used as the alkaline activator. Weighed sodium hydroxide particles were slowly added to a certain amount of tap water, quickly stirred using a glass rod, and then poured into the mixture of unfired bricks after being cooled to room temperature.

### 2.2. Preparation of Unfired Bricks

We used the single factor test to determine the optimal scheme for the preparation of unfired bricks composed of the aeolian sand–loess composite. The proportional design is provided in Table 3. Then, we conducted 17 groups of tests (three parallel samples, see Table 4) to study the effects of the cement, fly ash, polypropylene fibers, and molding pressure on the physical properties of the unfired bricks, and to obtain their optimal mixing ratio and molding pressure. It is worth clarifying that the cementitious materials in Table 4 refer to cement and fly ash.

**Figure 5:**
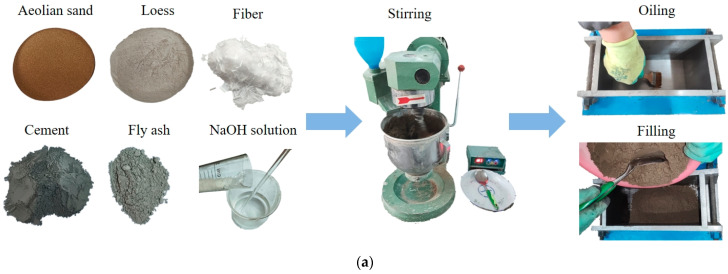

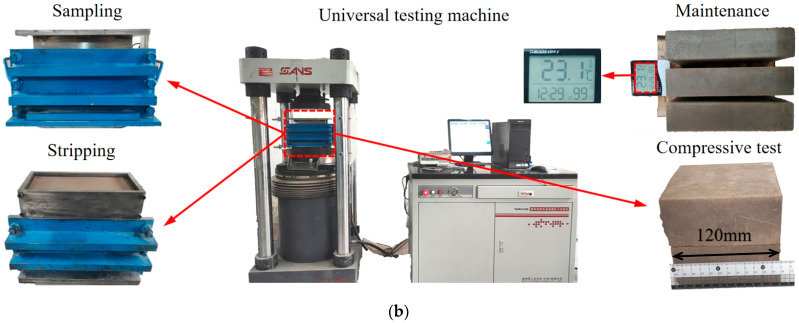
Process of preparing unfired brick samples. (**a**) Preprocess. (**b**) Sample preparation.

The process of the preparation of the unfired bricks Is shown in Figure 5. The raw materials for them were loaded into the mixer according to the preset ratios shown in Table 3 and fully mixed for 3 min. A fixed proportion of water was then added to the mixture, and it was continuously stirred for 5 min. The mixed wet mixture was loaded into a steel mold with dimensions of 240 mm × 115 mm × 158 mm. This mold was pressed by a universal testing machine to a specified pressure for a specified time, after which it was demolded; the pressure was maintained for 1 min after reaching the specified pressure. After demolding, the sample was placed in a curing box, and was naturally cured for 7 d and 14 d at a curing temperature of (21 ± 2) °C and a relative humidity of 95% ± 5%. The opening of the box was covered with a plastic film to prevent the evaporation of a large amount of water.

### 2.3. Methods

The test materials in group A included aeolian sand and loess. The unconfined compressive strength of the materials in group A was tested with reference to the Standard for Geotechnical Testing GB/T50123-2019 [41] and using a cylindrical sample with a size of Φ39.1 × 80 mm. Their stress–strain curve was plotted based on the results.

The test samples of groups B, C, D, and E were all samples of unfired bricks with dimensions of 240 mm × 115 mm × 158 mm. Their compressive strengths, water absorptions, coefficients of softening, densities, and resistances to frost were tested with reference to the Test Methods for Wall Bricks GB/T 2542-2012 [42].

The compressive strength ${\;R}_{P}$ of the bricks was tested using a universal testing machine and was calculated using Equation (1):

(1) $$R_{P}=\frac{P}{L\times B}$$

where P is the maximum failure-inducing load, and L and B are the length and width of the unfired bricks, respectively.

The bulk density was calculated as follows:

(2) $$\rho =\frac{m}{V}$$

where *m* is the dry mass of the sample that was measured using an electronic scale with an accuracy of 0.01, and V is the volume of the unfired brick sample that was empirically measured.

The water absorption $W_{24}$ of the bricks was calculated using Equation (3). The unfired brick sample was first dried to a constant mass in a blast drying oven at 110 °C, and the dried sample was then immersed in water at 20 °C for 24 h. Following this, the sample was weighed once its surface had been wiped dry.

(3) $$W_{24}=\frac{m_{24}-m_{0}}{m_{0}}\times 100$$

where $m_{0\;}$ is the dry mass of the sample, and $m_{24}$ is its mass after having been immersed in water for 24 h.

The coefficient of softening $K_{f}$ was calculated using Equation (4). The unfired brick sample was placed in an oven and dried at 110 °C for 2 h. The dried sample was then immersed in water at room temperature for 4 d. The compressive strengths of the immersed samples after 4 d were compared with that of the sample that was air-dried for 72 h.

(4) $$K_{f}=\frac{R_{f}}{R_{0}}$$

where $R_{f}$ is the mean compressive strength of the sample after softening, and $R_{0}$ is the mean strength of the contrast sample.

The unfired brick sample was placed in the oven and dried at 110 °C for 2 h. The dried sample was then immersed in water at ambient temperature for 24 h, taken out and dried, placed in a refrigerator at −20 °C for 5 h, and then immersed in water for 3 h. This constituted a freeze–thaw cycle for the test of its resistance to frost. We used 15 freeze–thaw cycles.

(5) $$P_{m}=\frac{P_{0}-P_{1}}{P_{0}}\times 100$$

(6) $$G_{m}=\frac{m_{0}-m_{1}}{m_{0}}\times 100$$

where $P_{m}$ is the rate of loss of strength of the sample, $P_{0}$ is its strength before freezing and thawing, $P_{1}$ is the strength of the sample after freezing and thawing, $G_{m}$ is the rate of mass loss, $m_{0}$ is the dry mass of the sample before freezing and thawing, and $m_{1}$ is its dry mass after freezing and thawing.

## 3. Results

### 3.1. Physical Properties of Unfired Bricks

**Figure 6:**
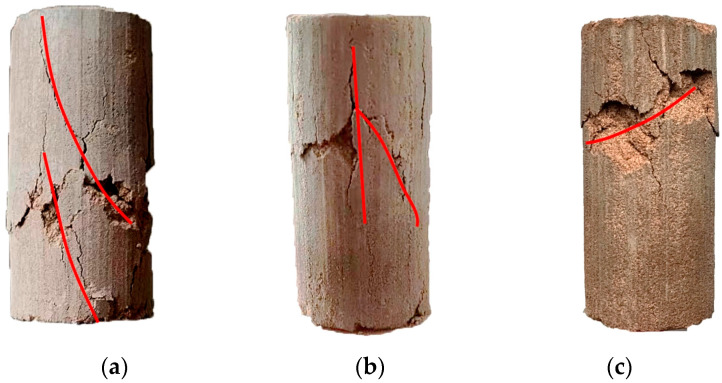
Modes of shear failure of composite samples with different ratios of aeolian sand and loess. (**a**) 1:1. (**b**) 2:1. (**c**) 3:1.

**Figure 7:**
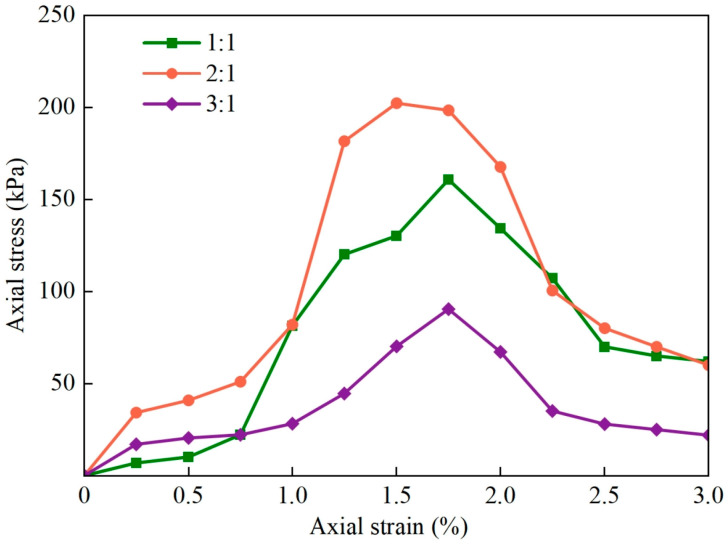
Curves of axial stress versus axial strain of the samples.

Modes of the shear failure of the samples in group A are shown in Figure 6. The samples underwent shear failure, with apparent longitudinal and oblique cracks. They did not reach their maximum compactness when the ratio of aeolian sand to loess was 1:1. The particles of aeolian sand and loess were fully embedded, with tight mutual occlusion and the lowest porosity, when the ratio of aeolian sand to loess was 2:1. The sample exhibited the maximum compactness and the highest strength at this ratio. When the ratio of aeolian sand to loess was 3:1, the loess particles could not fill the pores in the sand particles, and this caused the sample to become weak. The curve of the axial stress versus the strain of the unfired brick sample (Figure 7) showed that it had the best mechanical properties when the ratio of aeolian sand to loess was 2:1.

**Figure 8:**
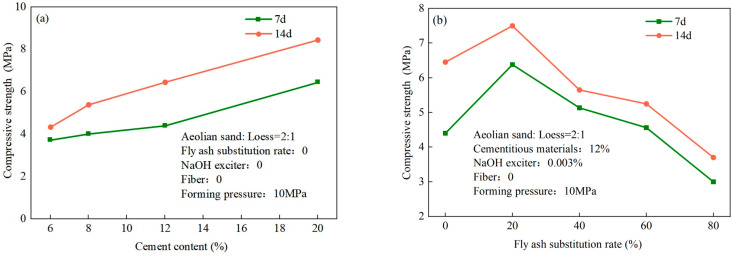

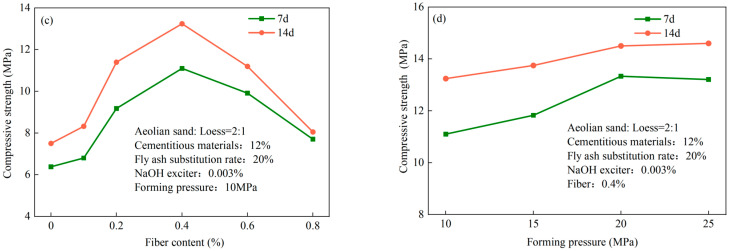
Effects of different conditions of preparation on the compressive strength of unfired bricks: (**a**) cement content, (**b**) rate of substitution of fly ash, (**c**) fiber content, and (**d**) forming pressure.

The effects of the different conditions of preparation on the compressive strength of the unfired bricks are illustrated in Figure 8. Figure 8a shows that their compressive strength increased gradually with the cement content because such gel substances as C-S-H and Ca(OH)_2_, which were generated through the hydration of cement, filled small voids still present in the mixed particles of aeolian sand and loess after compaction. This increased the cohesion between the aggregates. The compressive strength of the sample reached 8.45 MPa after 14 d when its cement content was 20%. Given that the use of cement leads to environmental pollution and incurs a high cost, we chose 12% as the optimal cement content. The compressive strength of the sample was 6.54 Mpa in this case. As the rate of fly ash substituted into the sample increased, its compressive strength first increased and then decreased, with a highest value of 7.50 Mpa after 14 d when the rate of substituted fly ash was 20% (Figure 8b). Under alkaline excitation, the fly ash fully reacted with Ca(OH)_2_ to form a large number of acicular and strip C-S-H gel substances that filled the internal pores and rendered the unfired brick more compact [43]. As the rate of substitution of fly ash was gradually increased, the number of fly ash particles increased to enhance the fluidity of the aggregate and made it difficult to form the unfired bricks. Moreover, most of the fly ash did not participate in the hydration reaction and hindered the hydration of cement to increase the internal porosity and significantly reduce the strength of the unfired bricks [44]. The effects of different fiber contents on the compressive strength of the unfired bricks are shown in Figure 8c. As the content of the polypropylene fibers was increased, the compressive strength of the bricks first increased and then decreased. When the polypropylene fiber content was 0.4%, their strength was the highest at 13.24 Mpa, and decreased gradually when the fiber content was further increased. This is because the added polypropylene fibers, together with the aggregate and the gel substances, generated mechanical occluding and frictional forces. When the unfired bricks were subjected to an axial load, the polypropylene fibers prevented the expansion of internal transverse cracks in them. Moreover, the fibers were randomly dispersed to form a spatial network that inhibited the deformation of soil and enhanced the compressive strength of the unfired bricks [45]. When the fiber content exceeded 0.4%, fiber agglomeration occurred owing to the uneven distribution of fibers in the unfired bricks. Moreover, the uniformity of the aggregate worsened and enabled the unfired bricks to generate more weak surfaces to reduce their strength [45].

**Figure 9:**
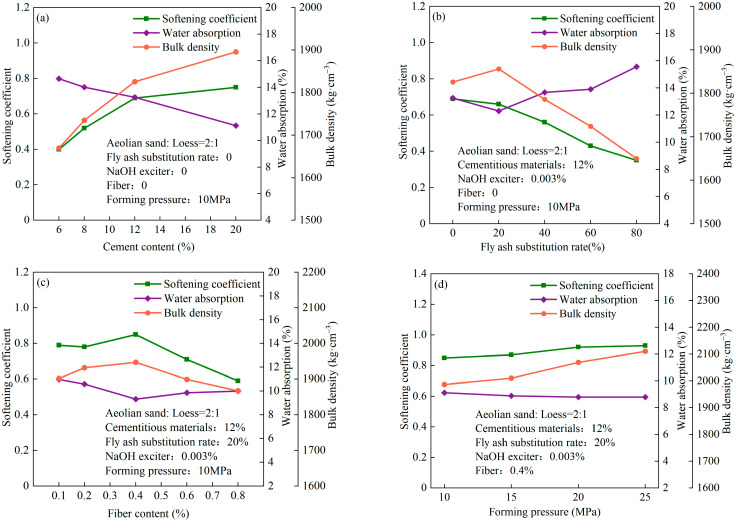
Effects of different conditions of preparation on the water resistance and bulk density of the unfired bricks: (**a**) cement content, (**b**) rate of substitution of fly ash, (**c**) fiber content, and (**d**) forming pressure.

The effects of the different conditions of preparation on the water resistance and bulk density of the unfired bricks are shown in Figure 9. It is clear from it that the water absorption and coefficient of softening of the unfired bricks were negatively correlated. Owing to the high density of aeolian sand and loess, and their relatively large masses in the aggregate of unfired bricks (84.15%), the bulk density of the latter was not significantly influenced by different conditions of preparation. Figure 9a shows that as the cement content increased, the cement and its products of hydration continued to increase in volume, thus increasing the bulk density of the bricks and reducing their water absorption to increase their coefficient of softening. Figure 9b shows that the addition of an appropriate amount of fly ash increased the compactness of the bricks. When the rate of substitution of fly ash was 20%, the rate of water absorption was 12.3%, and the coefficient of softening was 0.66. With a gradual increase in the rate of substitution of fly ash, the porosity of the bricks increased, resulting in an increase in their rate of water absorption, a decrease in their coefficient of softening, and thus a deterioration in their water resistance. It is evident from Figure 9c that the added fibers were conducive to the water resistance of the unfired bricks. When a small amount of fibers was added, the fibers and aggregate particles were in complete contact and tightly bonded to reduce internal porosity, thereby reducing the rate of water absorption and increasing the softening coefficient [20,46]. When the fiber content of the bricks was 0.4%, their rate of water absorption reached the minimum value of 9.32%, the coefficient of softening reached the maximum value of 0.85, and the bulk density was 1947 kg/m^3^. Excessive fiber agglomeration led to a large number of uneven pores inside the unfired bricks, resulting in increased rates of water absorption, coefficient of softening, and bulk density.

Forming pressure is one of the key preparation conditions for unfired bricks, and Figure 8d and Figure 9d indicate the effects of different forming pressures on the compressive strength, softening coefficient, water absorption, and bulk density of unfired bricks. Figure 8d shows that the compressive strength of the unfired bricks gradually increases and eventually tends to be constant as the forming pressure increases. At a forming pressure of 20 MPa, the compressive strength of the unfired bricks after 14 d was 14.50 MPa, while the water absorption reached a minimum value of 8.8% and the softening coefficient was 0.92. Moreover, the mixture of unfired bricks was compacted, and the C-S-H and other gel substances fully bonded the aggregate to lead to small inter-granular pores in it and its highest strength. When the forming pressure was 25 MPa, there was no significant increase in the strength of the unfired bricks, but their bulk density reached a maximum value of 2110 kg/m^3^, water absorption decreased, and softening coefficient increased (see Figure 9d). However, the more forming pressure there is, the higher the demands on the forming tool. Therefore, 20 MPa is the optimal forming pressure for comprehensive cost considerations.

Based on the above analysis, we obtained the following optimal mixing ratios of the unfired bricks: 56.10% of aeolian sand, 28.05% of loess, 9.17% of cement, 2.40% of fly ash, and 0.4% of polypropylene. The optimal forming pressure is 20 MPa.

### 3.2. Freeze–Thaw Cycle Test of Unfired Bricks

**Figure 10:**
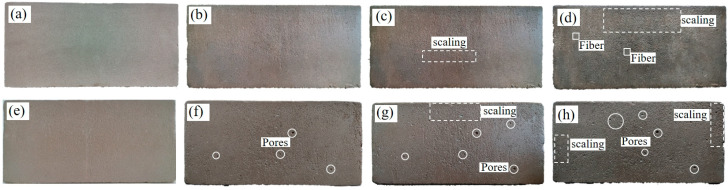
Diagrams of the evolution of the appearance of unfired bricks under different numbers of freeze–thaw cycles. Fiber content of 0.4%: (**a**) 0 cycles, (**b**) 5 cycles, (**c**) 10 cycles, and (**d**) 15 cycles; Fiber content of 0%: (**e**) 0 cycles, (**f**) 5 cycles, (**g**) 10 cycles, and (**h**) 15 cycles.

**Figure 11:**
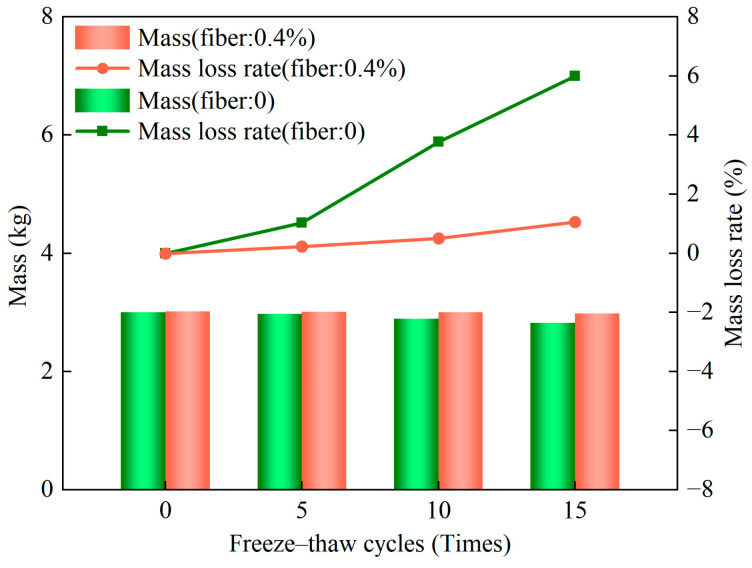
Evolution of the rate of mass loss under different numbers of freeze–thaw cycles.

The rates of losses in the mass and strength of the unfired bricks as obtained by the freeze–thaw cycle test represent the indices of their resistance to frost. Diagrams of the appearance of deterioration in the samples with a fiber content of 0.4% after 0, 5, 10, and 15 freeze–thaw cycles are shown in Figure 10a–d, respectively. With an increase in the number of freeze–thaw cycles, some of the aggregate were shed from the surface of the sample to expose some fibers. The appearance of deterioration in the sample with zero fiber content was more prominent, with a large number of pores, the shedding of a large amount of aggregate, and block defects around the sample (Figure 10e–h). The relationship between the rate of loss of mass of the unfired bricks and the number of freeze–thaw cycles is illustrated in Figure 11. After 5, 10, and 15 freeze–thaw cycles, the rates of loss of mass of the unfired bricks with a fiber content of 0.4% were 0.23%, 0.5%, and 1.06%, respectively, while those of bricks with zero fiber content were 1.03%, 3.77%, and 6%, respectively. That is, as the number of freeze–thaw cycles increased, the shedding of the aggregate gradually became significant, and the rates of loss of mass of both increased. However, the rate of loss of mass of the former was much smaller than that of the latter. Accordingly, the results of the rate of loss of mass quantitatively verified the results of the evolution in the appearance of the brick samples [47]. The fiber content was 0.4% for samples (a)–(d) and zero for samples (e)–(h).

**Figure 12:**
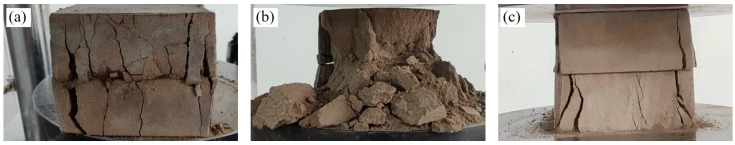
The modes of failure of frozen–thawed samples and non-frozen–thawed samples. (**a**) Fiber content, 0.4%, (**b**) zero fiber content, and (**c**) non-frozen–thawed sample with a fiber content of 0.4%.

**Figure 13:**
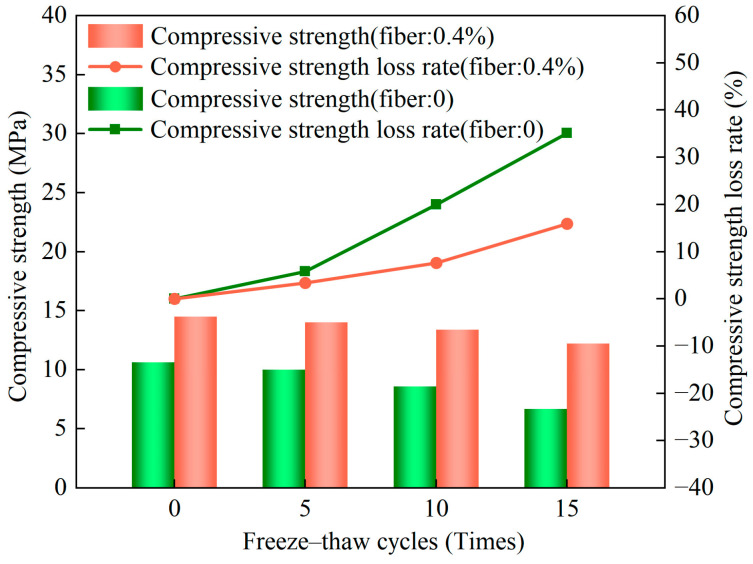
Evolution of the rate of loss in strength under different numbers of freeze–thaw cycles.

The modes of failure of the frozen–thawed and non-frozen–thawed samples in the tests of compressive strength are shown in Figure 12. When a sample was subjected to an axial load, longitudinal cracks first appeared in it. As the vertical stress was gradually increased, the polypropylene fibers inside the sample participated in inhibiting the formation of transverse cracks in it [45,48,49], and longitudinal cracks were thus mostly observed when the sample was damaged (Figure 12c). However, the encapsulation between the fibers and the aggregate in the sample worsened after freezing and thawing, the transverse tensile effect was negligibly small, the structure was loose, and the longitudinal and transverse cracks were interlaced (Figure 12a). Figure 12b shows that internal cracks in the sample developed rapidly due to the absence of the tensile effect of the fibers, and it collapsed when the axial load reached the preset limit. The relationship between the rate of loss in the strength of the unfired bricks and the number of freeze–thaw cycles is illustrated in Figure 13. After 5, 10, and 15 freeze–thaw cycles, the rates of loss in the strength of unfired bricks with a fiber content of 0.4% were 3.38%, 7.66%, and 15.93%, respectively, while the rates of loss in the strength of bricks with zero fiber content were 5.83%, 19.96%, and 36.15%, respectively. That is, as the number of freeze–thaw cycles increased, the rate of loss in the strength of bricks with a fiber content of 0.4% increased exponentially while that of bricks with zero fiber content increased by three to four times. Adding an appropriate number of fibers thus significantly improved the resistance of the unfired bricks to frost.

### 3.3. Mechanism of Strength Formation of Unfired Bricks

#### 3.3.1. XRD

**Figure 14:**
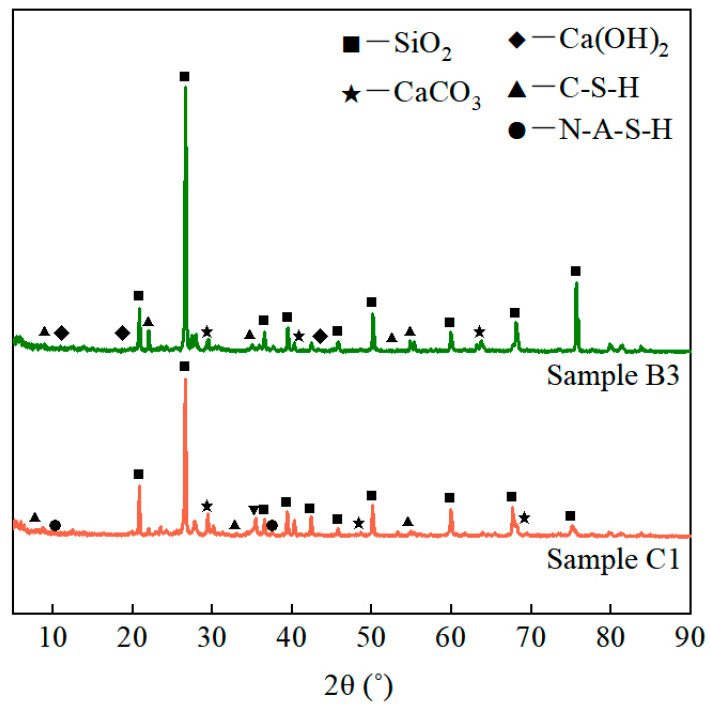
XRD patterns of unfired bricks (cement content of 12% and rate of substitution of fly ash of 20%).

To investigate the mechanism of the strength formation of the unfired bricks, we subjected them to XRD analysis under different mixing ratios, as shown in Figure 14. We considered two kinds of samples, with a cement content of 12% (of which 20% was replaced with fly ash). Their phase compositions were approximately the same, with a consistent peak strength of SiO_2_ because it is the main compound in aeolian sand, had a high mass ratio, and acted as the skeleton of unfired bricks. The phase composition of the former involved the products of the hydration of cement, such as Ca(OH)_2_ and C-S-H, while the latter was rehydrated with fly ash to generate a sodium silico-aluminate gel (N-A-S-H) and more C-S-H due to the addition of fly ash. This led to a fuller encapsulation of the particles of the aggregate. The structure of the unfired bricks was thus compact and significantly improved their strength.

#### 3.3.2. SEM

**Figure 15:**
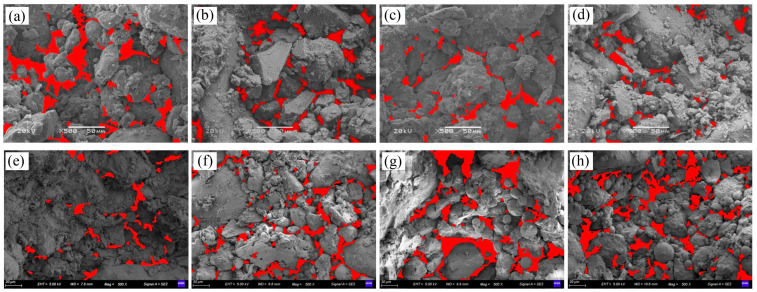
Pore distribution of the samples under different mixing ratios (500×). (**a**–**d**): The cement contents were 6%, 8%, 12%, and 20%. (**e**–**h**): The rates of substitution of fly ash were 20%, 40%, 60%, and 80%. The red regions in this figure are pores.

**Figure 16:**
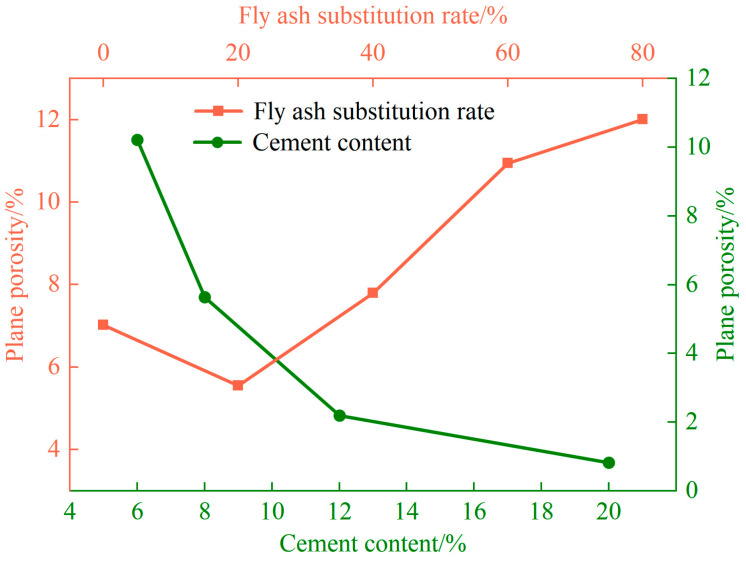
Effects of different conditions of preparation on the surface porosity of the unfired bricks.

**Figure 17:**
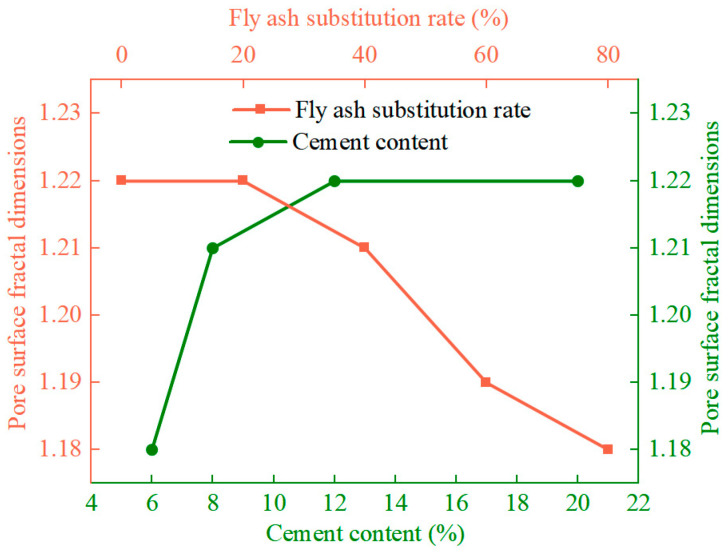
Effects of different conditions of preparation on the pore surface fractal dimensions of the unfired bricks.

Microscopic images of the samples at different magnifications were obtained through SEM tests. The images were preprocessed using Image Pro Plus 6.0 (Figure 15), and the surface porosity of the samples was calculated (Figure 16), as well as the pore surface fractal dimensions (Ds), which are a quantitative description of the roughness and complexity of the internal pore structure (Figure 17) [50,51,52]. The surface porosity *P* is the ratio of the pore area of soil to the total area of the observation field (can be calculated from Equation (7)) and reflects the compactness of the soil:

(7) $$P=\frac{A_{e}}{A}$$

where $A_{e}$ is the total area of pores in the image, and *A* is the total area of the image itself.

Figure 16 shows that the porosity of the surfaces of the unfired bricks decreased with an increase in their cement content, increased with the rates of the substitution of fly ash and water absorption, and was negatively correlated with their compressive strength, coefficient of softening, and bulk density. From Figure 17, it can be seen that Ds increases with the increase in cement content, decreases with the increase in the fly ash substitution rate and water absorption, and is positively correlated with its compressive strength. As the cement content was increased, the volume of the products of hydration increased, and led to the encapsulation of the aggregate particles that filled the internal pores to yield a denser structure (Figure 15a–d). The surface porosity decreases, and the pore structure is rougher and more complex than the larger Ds’ is.

Figure 15e–h show that the excessive fly ash inhibited the hydration of the cementitious materials, leading to the significant separation of the aggregate and increased porosity of the samples. The more homogeneous the pore structure is, the smaller the Ds is. Combined with the change curve of compressive strength, it can be found that the compressive strength increases with the increase of Ds, indicating that Ds is a key parameter for predicting cement-based unfired bricks [50].

**Figure 18:**
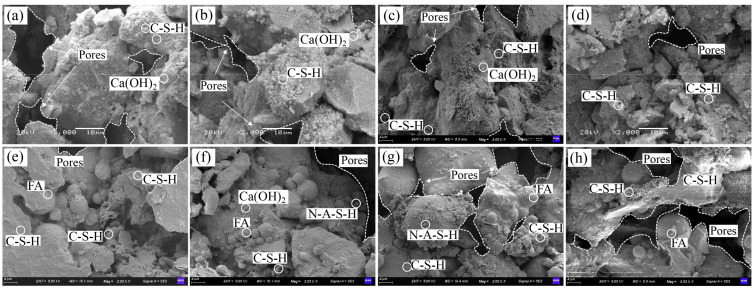
Pore distributions of the test samples under different mixing ratios (2000×). (**a**–**d**): The cement contents of the samples were 6%, 8%, 12%, and 20%, respectively; (**e**–**h**): The rates of substitution of fly ash were 20%, 40%, 60%, and 80%, respectively.

The SEM images of the samples at a magnification of 2000× are shown in Figure 18. According to Figure 18a–d, the hydration of cement generated an increasing volume of flocculent C-S-H (I)-type gels with an increasing cement content to fill in the pores in the aggregate and reduce its porosity. When the content of fly ash was low, it reacted fully with the alkaline substances to form a high-density C-S-H (II)-type gel. With an increase in the content of the fly ash, only a small part of its particles and aggregates were encapsulated by the gel, leading to unfired bricks with a high porosity, loose structure, and reduced strength [53,54].

**Figure 19:**
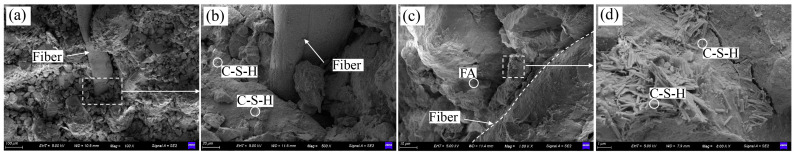
Microstructures of unfired bricks with optimal mixing ratio: (**a**) 100×; (**b**) 500×; (**c**) 1000×; (**d**) 8000×.

**Figure 20:**
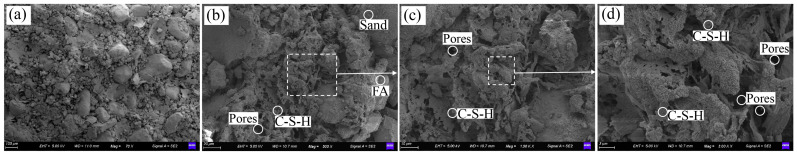
SEM images of softened sample: (**a**) 100×; (**b**) 500×; (**c**) 1000×; (**d**) 3000×.

Figure 19 shows that the polypropylene fibers were embedded into the particles of aeolian sand and loess as well as the gel materials, and the fibers and aggregates were connected by C-S-H. A large number of needle columnar C-S-H particles were observed through an electron microscope at a magnification of 8000×. The SEM images (Figure 20) of the softened samples revealed many pores and fragmentary structures inside the samples because they had been saturated in water for a long time. The water content of the pores increased, their structure was further damaged by the water pressure, and they became larger. Moreover, the connection between the aggregate and the gel eroded, leading to a reduction in the strength of the unfired bricks. Moreover, a large number of C-S-H-type gel substances with lamellar and globular structures were accumulated and were observable through the electron microscope at a magnification of 3000×.

**Figure 21:**
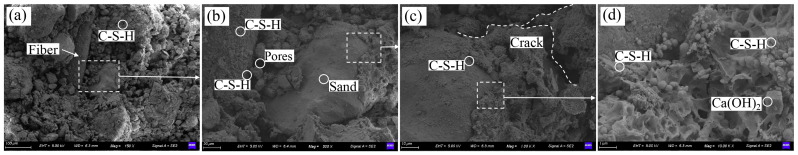
SEM images of frozen–thawed samples: (**a**) 100×; (**b**) 500×; (**c**) 3000×; (**d**) 10,000×.

The frozen–thawed samples were subjected to SEM analysis (Figure 21). After 15 freeze–thaw cycles, a large amount of the exfoliated gel wrapped around the sand particles, while the fibers and the aggregate were connected, and a large number of cracks appeared between the aggregates (Figure 21c). Figure 21d shows that a large amount of the gel material formed short columnar, globular, and honeycomb structures due to erosion induced by the freeze–thaw cycles, and these areas had low strength. The strength of the frozen–thawed samples decreased mainly because the pressure, which was generated once the moisture inside the pores of the unfired bricks was converted into ice, adversely affected the pore structure during freezing, and led to an increase in the pore volume. During thawing, the pore structure increased in size due to freezing, and this caused more water to migrate internally to fill the pores. After one freeze–thaw cycle, the increase in water in the pores and their volume accelerated the destruction of the unfired bricks, resulting in an increase in porosity and the further development of cracks. Finally, the strength of the unfired bricks decreased sharply.

## 4. Conclusions and Discussion

The results of this study show that it is feasible to prepare unfired bricks from aeolian sand and loess. By exploring the effects of different mixing ratios and molding pressures on the physical properties of the unfired bricks, we found that an increase in their cement content improved their compressive strength, coefficient of softening, and bulk density while reducing their absorption of water. The compressive strength and coefficient of softening of the unfired bricks decreased with an increase in their fly ash content, but the amount of water absorbed by them increased. An appropriate fiber content further improved their compressive strength and coefficient of softening and reduced the rate of water absorption. In contrast, an excessively large fiber content reduced their compressive strength and coefficient of softening and increased the rate of water absorption. The optimal mixing ratio of aeolian sand–loess in the unfired bricks was as follows (mass): aeolian sand–loess–cement–fly ash–polypropylene fiber–alkali exciter–water = 56.10:28.05:9.17:2.40:0.4:0.003:4.24 under a forming pressure of 20 MPa. The composite of unfired brick prepared using the optimal mixing ratio had a compressive strength of 14.5 MPa after 14 d, rate of water absorption of 8.8%, coefficient of softening of 0.92, and rates of loss in strength and mass of 15.93% and 1.06%, respectively. These satisfy the requirements of the strength grade of MU10–MU15 and can be used for non-load-bearing walls.

(1) During high-pressure pressing, the aeolian sand, loess, and fiber were in complete contact with the products of the hydration of the cementitious materials, and the bulk density and compactness of the cementitious bricks increased with the molding pressure. That is, the area of contact between the aggregate and the gel increased to enhance cementation within the aggregate.
(2) With an increase in the cement content, the volume of the products of hydration in the sample gradually increased, resulting in an increase in the compactness and resistance of the bricks to pressure. The gel substances generated by the fly ash under the action of the alkali activator were tightly connected with the particles of aeolian sand and the loess skeleton and filled the space between the particles to reduce porosity. When the bricks were cured at a temperature of (21 ± 2) °C and a relative humidity of 95% ± 5%, the products of hydration, C-S-H, sodium aluminosilicate hydrate (N-A-S-H), and other gel substances inside the unfired bricks gradually increased in volume to fill the internal pores, increased the compactness of the bricks, reduced their porosity, and improved their compressive strength.
(3) An appropriate amount of the polypropylene fiber was in complete contact with the aggregate and gel, and this increased the mechanical occluding force. The spatial network thus formed and inhibited the deformation of soil and increased the capability of the bonding of the unfired brick aggregates. Moreover, the compressive strength of the unfired bricks increased under an axial load.

Aeolian sand formed the main skeleton (56.10 wt%) of the unfired bricks, and provided support for them, but also increased their weight owing to the high density of the aeolian sand. However, their bulk density (1670–1947 kg/m^3^) was lower than that of other cement-based unfired bricks [55,56]. According to the strength-related requirements of standard GB/T5101-2017 [57], the strength of the unfired bricks composed of the aeolian sand–loess composite was 95% of that of bricks of strength grade MU15, and thus satisfied the requirements of MU10–MU15 such that they can be used for non-load-bearing walls. The unfired bricks could also be used in humid and cold environments owing to their suitable resistance to water and frost.

The main raw materials of the unfired bricks, aeolian sand and loess, are widely available. In contrast to other types of unfired bricks, these bricks did not require the processing of recycled raw materials into aggregates [11,16]. Moreover, they did not require a high-temperature environment for curing [58], which would have reduced their cost.

## Figures and Tables

**Table 1:** Chemical composition of aeolian sand and loess.

Content/%	SiO_2_	NaAlSiO₈	(K,Na)(Fe^3+^,Al,Mg)_2_(Si,Al)_4_O_10_(OH)_2_	Kal_2_(AlSi_3_O_10_)(OH)_2_
Aeolian Sand	51.0	19.4	3.3	19.4
Loess	40.0	23.0	3.2	14.6
**Content/%**	**CaCO_3_**	**Fe_2_O_3_**	**Al_2_Si_2_O_5_(OH)_4_**	**CaMg(CO_3_)_2_**
Aeolian Sand	0.4	2.6	3.9	-
Loess	10.7	1.2	3.8	3.5

**Table 2:** Chemical composition of the cementitious materials.

Content/%	SiO_2_	Al_2_O_3_	CaO	SO_3_	Fe	Cl^−^	NaOH	CaO_2_	MgO	TiO_2_	Na_2_O
Cement	45.1	24.2	5.6	2.1	0.85	0.015	1.2	0.85	-	-	-
Fly ash	20.58	5.64	62.25	3.18	3.95	-	-	-	2.48	0.32	0.36

**Table 3:** Scheme for aeolian sand–loess composites.

Number	Sample	Aeolian Sand–Loess
A	A1	1:1
	A2	2:1
	A3	3:1

**Table 4:** Scheme for the preparation of unfired bricks.

Number	Sample	Aeolian Sand–Loess	Cementitious Material/%	Fly Ash-to-Cement Replacement Ratio/%	NaOH Exciter/%	Fiber/%	Forming Pressure/Mpa
B	B1	2:1	6	0	0	0	10
	B2		8				
	B3		12				
	B4		20				
C	C1	2:1	12	20	0.003	0	10
	C2			40			
	C3			60			
	C4			80			
D	D1	2:1	12	20	0.003	0.1	10
	D2					0.2	
	D3					0.4	
	D4					0.6	
	D5					0.8	
E	E1	2:1	12	20	0.003	0.4	10
	E2						15
	E3						20
	E4						25

## Data Availability

Data are contained within the article.

## References

[1] The State Forestry Administration of the People’s Republic of China (2018). Atlas of Sandy Deserts in China.

[2] Rosenfeld D., Rudich Y., Lahav R. (2001). Desert dust suppressing precipitation: A possible desertification feedback loop. Proc. Natl. Acad. Sci. USA.

[3] Raffaele L., van Beeck J., Bruno L. (2021). Wind-sand tunnel testing of surface-mounted obstacles: Similarity requirements and a case study on a Sand Mitigation Measure. J. Wind Eng. Ind. Aerodyn..

[4] Aili A., Xu H., Xu Q., Liu K. (2023). Aeolian dust movement and deposition under local atmospheric circulation in a desert-oasis transition zone of the northeastern Taklimakan desert. Ecol. Indic..

[5] Roberts S.F., Duperret J.M., Li X., Wang H., Koditschek D. (2014). Desert RHex Technical Report: Tengger Desert Trip.

[6] Elipe M.G.M., López-Querol S. (2014). Aeolian sands: Characterization, options of improvement and possible employment in construction—The State-of-the-art. Constr. Build. Mater..

[7] Pye K., Tsoar H. (2008). Aeolian Sand and Sand Dunes.

[8] Bunce C., Smalley I., Stevens T., Assadi-Langroudi A. (2022). Loess in Britain and Ireland: Formation, modification and environmental significance, a review in memory of John Catt (1937–2017). Proc. Geol. Assoc..

[9] Pye K. (1995). The nature, origin and accumulation of loess. Quat. Sci. Rev..

[10] Assadi-Langroudi A., Ng’ambi S., Smalley I. (2018). Loess as a collapsible soil: Some basic particle packing aspects. Quat. Int..

[11] Seco A., Omer J., Marcelino S., Espuelas S., Prieto E. (2018). Sustainable unfired bricks manufacturing from construction and demolition wastes. Constr. Build. Mater..

[12] Amhadi T.S., Assaf G.J. (2021). Improvement of Pavement Subgrade by Adding Cement and Fly Ash to Natural Desert Sand. Infrastructures.

[13] Al-Aghbari M.Y., Mohamedzein Y.E.-A., Taha R. (2009). Stabilisation of desert sands using cement and cement dust. Proc. Inst. Civ. Eng.—Ground Improv..

[14] Al-Harthy A.S., Halim M.A., Taha R., Al-Jabri K.S. (2007). The properties of concrete made with fine dune sand. Constr. Build. Mater..

[15] Damene Z., Goual M.S., Houessou J., Dheilly R.M., Goullieux A., Quéneudec M. (2018). The use of southern Algeria dune sand in cellular lightweight concrete manufacturing: Effect of lime and aluminium content on porosity, compressive strength and thermal conductivity of elaborated materials. Eur. J. Environ. Civ. Eng..

[16] Maierdan Y., Haque M.A., Chen B., Maimaitiyiming M., Ahmad M.R. (2020). Recycling of waste river sludge into unfired green bricks stabilized by a combination of phosphogypsum, slag, and cement. Constr. Build. Mater..

[17] Netterberg F., Elsmere D. (2015). Untreated aeolian sand base course for low-volume road proven by 50-year old road experiment. J. S. Afr. Inst. Civ. Eng..

[18] Wang W.J., Gan Y.X., Kang X. (2021). Synthesis and characterization of sustainable eco-friendly unburned bricks from slate tailings. J. Mater. Res. Technol.-JMRT.

[19] Arias-Trujillo J., Matías-Sanchez A., Cantero B., López-Querol S. (2023). Mechanical stabilization of aeolian sand with ceramic brick waste aggregates. Constr. Build. Mater..

[20] Yang X., Hu Z., Wang Y., Wang X. (2024). Aeolian sand stabilized by using fiber- and silt-reinforced cement: Mechanical properties, microstructure evolution, and reinforcement mechanism. Constr. Build. Mater..

[21] Xue Z., Zhang Y., Luo J., Yan C., Emmanuel M., Jia X. (2024). Analysis of compressive strength, durability properties, and micromechanisms of solidified loess using industrial solid waste: Slag–white mud–calcium carbide residue. J. Build. Eng..

[22] Yang J.L., Li S.Y., Di H.G., Liu D.R., Wang X., Zhao J.Y. (2023). Experimental investigations on the physico-mechanical and microstructural properties of loess reinforced with anionic polyacrylamide. Constr. Build. Mater..

[23] Yang X., Hu Z.Q., Li L., Wang X.L., Zhou X. (2024). Strength properties, microstructural evolution, and reinforcement mechanism for cement-stabilized loess with silica micro powder. Case Stud. Constr. Mater..

[24] Golley J. (2007). China’s western development strategy and nature versus nurture. J. Chin. Econ. Bus. Stud..

[25] Niyomukiza J.B., Nabitaka K.C., Kiwanuka M., Tiboti P., Akampulira J. (2022). Enhancing Properties of Unfired Clay Bricks Using Palm Fronds and Palm Seeds. Results Eng..

[26] Noureddine O., Manssouri I., Sahbi H., Limami H., Khaldoun A. (2023). Rheological and physico-mechanical investigations on the destabilization of unfired clay bricks with almond husk additive by salt. Constr. Build. Mater..

[27] Jiménez-García E.d.J., Arellano-Vazquez D.A., Titotto S., Vilchis-Nestor A.R., Mayorga M., Romero-Salazar L., Arteaga-Arcos J.C. (2023). A low environmental impact admixture for the elaboration of unfired clay building bricks. Constr. Build. Mater..

[28] El-Yahyaoui A., Manssouri I., Noureddine O., Sahbi H., Khaldoun A. (2023). Physical and mechanical properties of unfired clay bricks with saw palmetto fibers additive as a construction material. Mater. Today: Proc..

[29] Gupta V., Chai H.K., Lu Y., Chaudhary S. (2020). A state of the art review to enhance the industrial scale waste utilization in sustainable unfired bricks. Constr. Build. Mater..

[30] Dai W., Zheng Y., Chen X., Cang D. (2018). Pressing process and coloring property of baking-free bricks made of molybdenum tailing and cement. Chin. J. Eng..

[31] Li Z.P., Liu X.M., Li Y., Ren Y.Y., Wang Y.G., Zhang W. (2020). Effects of sulfate on the mechanical performances and hydration characteristics of red mud based non-burnt brick. Constr. Build. Mater..

[32] Li Y.R., Shi W.H., Aydin A., Beroya-Eitner M.A., Gao G.H. (2020). Loess genesis and worldwide distribution. Earth-Sci. Rev..

[33] Khan I.H. (1982). Soil studies for highway construction in arid zones. Eng. Geol..

[34] Abu-Zeid M.M., Baghdady A.R., El-Etr H.A. (2001). Textural attributes, mineralogy and provenance of sand dune fields in the greater Al Ain area, United Arab Emirates. J. Arid. Environ..

[35] Baghdadi Z.A., Rahman M.A. (1990). The potential of cement kiln dust for the stabilization of dune sand in highway construction. Build. Environ..

[36] Albusoda B.S., Salem L., Salem K. (2012). Stabilization of dune sand by using cement kiln dust (CKD). J. Earth Sci. Geotech. Eng..

[37] De Weerdt K., Ben Haha M., Le Saout G., Kjellsen K.O., Justnes H., Lothenbach B. (2011). Hydration mechanisms of ternary Portland cements containing limestone powder and fly ash. Cem. Concr. Res..

[38] Lothenbach B., Le Saout G., Gallucci E., Scrivener K. (2008). Influence of limestone on the hydration of Portland cements. Cem. Concr. Res..

[39] Liu J., Dong Y.C., Dong X.F., Hampshire S., Zhu L., Zhu Z.W., Li L.L. (2016). Feasible recycling of industrial waste coal fly ash for preparation of anorthite-cordierite based porous ceramic membrane supports with addition of dolomite. J. Eur. Ceram. Soc..

[40] Chang C.D., Zoback M.D., Khaksar A. (2006). Empirical relations between rock strength and physical properties in sedimentary rocks. J. Pet. Sci. Eng..

[41] (2019). Standard for Geotechnical Testing.

[42] (2012). GB/T 2542Test Methods for Wall Bricks.

[43] Cho Y.K., Jung S.H., Choi Y.C. (2019). Effects of chemical composition of fly ash on compressive strength of fly ash cement mortar. Constr. Build. Mater..

[44] Wei C., Pan X.L., Pei J.N., Lyu Z.Y., Yu H.Y. (2023). Preparation and characterization of unfired lightweight bricks using dealkalized calcium silicate residue from low-calcium sintering red mud. J. Cent. South Univ..

[45] Ruan B., Zhang J., Ding H., Yuan Z., Nie R. (2022). Experimental study on unconfined compressive strength and microstructure of cemented aeolian sand reinforced with basalt fiber. Journal of Rail Way Science and Engineering.

[46] Teixeira R.S., Santos S.F., Christoforo A.L., Savastano H., Lahr F.A.R. (2019). Extrudability of cement-based composites reinforced with curauá (*Ananas erectifolius*) or polypropylene fibers. Constr. Build. Mater..

[47] Yang J.L., Li S.Y., Di H.G., Liu D.R., Wang X., Zhao Y.C. (2024). Influence of anionic polyacrylamide on the freeze-thaw resistance of silty clay. Cold Reg. Sci. Tech..

[48] Mashayekhi A., Hassanli R., Zhuge Y., Ma X., Chow C.W. (2024). Synergistic effects of fiber hybridization on the mechanical performance of seawater sea-sand concrete. Constr. Build. Mater..

[49] Gani A., Ibrahim M., Ulmi F., Farhan A. (2024). The influence of different fiber sizes on the flexural strength of natural fiber-reinforced polymer composites. Results Mater..

[50] Qin Q., Meng Q., Yi P., Wu K., Wang C. (2023). Investigation on the rheology, self-shrinkage, pore structure, and fractal dimension of coral powder-cement slurry. J. Build. Eng..

[51] Dathe A., Eins S., Niemeyer J., Gerold G. (2001). The surface fractal dimension of the soil–pore interface as measured by image analysis. Geoderma.

[52] Dathe A., Thullner M. (2005). The relationship between fractal properties of solid matrix and pore space in porous media. Geoderma.

[53] Scherb S., Maier M., Köberl M., Beuntner N., Thienel K.-C. (2024). Reaction kinetics during early hydration of calcined phyllosilicates in model cement systems. Cem. Concr. Res..

[54] Deschner F., Winnefeld F., Lothenbach B., Seufert S., Schwesig P., Dittrich S., Goetz-Neunhoeffer F., Neubauer J. (2012). Hydration of Portland cement with high replacement by siliceous fly ash. Cem. Concr. Res..

[55] Xiong W., Chen Y., Xu J., Zhang Z., Liang C. (2023). Reuse of engineering waste soil and recycled fine aggregate to manufacture eco-friendly unfired clay bricks: Experimental assessment, data-driven modeling and environmental friendliness evaluation. Case Stud. Constr. Mater..

[56] Zhao H., Gou H.Y. (2021). Unfired bricks prepared with red mud and calcium sulfoaluminate cement: Properties and environmental impact. J. Build. Eng..

[57] (2017). Fired Common Bricks.

[58] Mezencevova A., Yeboah N.N., Burns S.E., Kahn L.F., Kurtis K.E. (2012). Utilization of Savannah Harbor river sediment as the primary raw material in production of fired brick. J. Environ. Manag..

